# Evaluating the Outcomes of the Capacity-Building Support From a Collaborative Network of Implementation Science Technical Assistance Hubs: Protocol for a Mixed Methods Evaluation

**DOI:** 10.2196/91374

**Published:** 2026-07-17

**Authors:** Sung-Jae Lee, Hannah Mestel, Carolyn M Audet, Bijal Balasubramanian, Nanette Benbow, Shruti Chandra, Carrie Geremia, Raul U Hernandez-Ramirez, Joyce L Jones, Olivia Keatley, Christopher Kemp, Christine M Khosropour, Robin Gaines Lanzi, Mary-Louise E Millett, Alexandra B Morshed, April C Pettit, Alec Powers, Borsika A Rabin, Jessica M Sales, Nicole A Stadnick, Debbie Humphries

**Affiliations:** 1 Psychiatry and Biobehavioral Sciences, David Geffen School of Medicine Division of Population Behavioral Health University of California, Los Angeles Los Angeles, CA United States; 2 Yale School of Public Health Yale University New Haven, CT United States; 3 Department of Health Policy Vanderbilt University Nashville, TN United States; 4 UTHealth Houston Institute for Implementation Science The University of Texas Health Science Center at Houston School of Public Health Houston, TX United States; 5 Department of Psychiatry and Behavioral Sciences, Feinberg School of Medicine Impact Institute Northwestern University Chicago, IL United States; 6 Impact Institute Northwestern University Chicago, IL United States; 7 Altman Clinical and Translational Research Institute Dissemination and Implementation Science Center Herbert Wertheim School of Public Health and Human Longevisty Science University of California San Diego La Jolla, CA United States; 8 Department of Medicine Division of Infectious Diseases Johns Hopkins Medicine Baltimore, MD United States; 9 Emory Center for AIDS Research Emory University Atlanta, GA United States; 10 Department of International Health Bloomberg School of Public Health Johns Hopkins University Baltimore, MD United States; 11 Department of Epidemiology University of Washington Seattle, WA United States; 12 Department of Health Behavior School of Public Health University of Alabama at Birmingham Birmingham, AL United States; 13 Behavioral, Social, and Health Education Sciences Department Rollins School of Public Health Emory University Atlanta, GA United States; 14 Vanderbilt University Medical Center Nashville, TN United States; 15 Department of Psychiatry Altman Clinical and Translational Research Institute Dissemination and Implementation Science Center, San Diego HIV Implementation Science Center, Child and Adolescent Services Research Center University of California San Diego La Jolla, CA United States

**Keywords:** HIV, Ending the HIV Epidemic in the US, implementation science, mixed methods evaluation, social network analysis, interviews, capacity building

## Abstract

**Background:**

Implementation science (IS) plays a critical role in translating research into real-world health outcomes. Few studies have evaluated models that provide technical assistance and other resources to build IS capacity. The Coordinating and Capacity-Building Hubs to Enhance the Science of HIV Implementation Research (CHESHIRE) network supports US-based HIV research awardees participating in the Ending the HIV Epidemic in the US (EHE) initiative.

**Objective:**

The objective of this study is to describe the evaluation protocol for CHESHIRE and assess the effect of the implementation of hub technical assistance activities on EHE-funded research team outcomes, including IS competencies, scientific collaboration, and research productivity.

**Methods:**

This protocol describes a mixed methods evaluation using qualitative interviews with EHE project leads (n=36); social network analysis of CHESHIRE-affiliated researchers and hub members (n=265); and secondary data analysis of National Institutes of Health (NIH) RePORTER, PubMed, and NIH Implementation Science Coordination Initiative EHE Project Final Progress Report Survey data (n=248). We will use descriptive analyses, network metrics, and thematic coding to describe outcomes following CHESHIRE implementation, including IS competencies, interinstitutional partnerships, and research productivity.

**Results:**

CHESHIRE coordinating center and hub activities were funded through NIH Center for AIDS Research and AIDS Research Center supplements beginning in 2019, with hubs funded between 2019 and 2024. Available survey data include EHE projects that completed the EHE Project Final Progress Report Survey between August 2021 and February 2025. As of January 2026, we have completed enrollment, with 265 network members in the final recruiting list. Data abstraction and analysis of the evaluation components are ongoing. Publication of findings is anticipated for December 2026. Evaluation findings will be disseminated after completion of data analysis. The findings will provide insights on whether structured IS support through CHESHIRE increases researchers’ IS competencies, interinstitutional partnerships, and research productivity.

**Conclusions:**

This evaluation will provide empirical evidence to guide the development and optimization of technical assistance hubs in public health research and inform their future evaluation of hub-based IS capacity-building models. Findings will inform strategies to optimize IS capacity building and accelerate the translation of evidence-based interventions into practice, especially in HIV prevention and treatment.

**International Registered Report Identifier (IRRID):**

DERR1-10.2196/91374

## Introduction

The Ending the HIV Epidemic in the US (EHE) initiative, launched by the US Department of Health and Human Services in 2019, aims to achieve a 90% reduction in new HIV infections nationwide by 2030 through a 4-pillar strategy: diagnose, treat, prevent, and respond [1]. As part of this national effort, the National Institutes of Health (NIH) has emphasized the importance of implementation science (IS) in bridging the gap between evidence-based interventions and their real-world application, particularly within underserved communities disproportionately affected by HIV.

Despite growing recognition of the importance of IS in public health, many researchers and practitioners lack formal training or structured opportunities to apply IS theory and methods in their work [2]. Studies have shown that capacity-building initiatives through mentorship, training, and consultations can enhance IS competencies, improve intervention fidelity, and support the sustainability of evidence-based practices [3,4]. However, few evaluations have rigorously assessed the impact of network-based IS technical assistance models.

Moreover, collaborative research networks play a critical role in advancing scientific discovery and dissemination. A robust body of literature underscores the importance of scientific social networks for academic productivity, knowledge diffusion, and interdisciplinary innovation [5]. However, how such networks evolve in response to structured technical assistance initiatives and whether they translate into measurable outcomes in HIV IS productivity remains underexplored.

To strengthen the application of IS methodologies and foster collaboration across the EHE landscape, the NIH funded the Coordinating and Capacity-Building Hubs to Enhance the Science of HIV Implementation Research (CHESHIRE) network. The network and planned evaluation have been described previously [6]. The CHESHIRE network, comprising 9 regional hubs and a central coordinating center (the HIV Implementation Science Coordination Initiative; ISCI), provides technical assistance, mentorship, and capacity building for HIV research teams receiving NIH EHE supplements. The network is grounded in the belief that centralized and regionally tailored IS support can accelerate the scale-up of proven interventions and result in equitable health outcomes [7-11].

This study protocol presents a comprehensive mixed methods evaluation of the CHESHIRE network, with three primary aims: to (1) assess changes in EHE-funded researchers’ IS competencies and use of IS tools; (2) examine the development and strengthening of scientific collaborations among those researchers; and (3) describe the productivity of EHE-funded researchers, including grants and publications, as potential outcomes of network engagement. Findings from this evaluation will inform future investments in capacity-building infrastructure and provide a model for scalable, networked approaches to IS support across public health domains.

This study protocol seeks to contribute to the broader goals of EHE and offer actionable guidance for enhancing the reach and effectiveness of HIV implementation research.

## Methods

### Study Design and Setting

This study uses a mixed methods evaluation design to describe outcomes following implementation of the CHESHIRE network. The evaluation includes 3 complementary components: qualitative interviews with EHE project leads; social network analysis (SNA) of CHESHIRE-affiliated researchers and hub members; and secondary analysis of survey, grant, and publication data. Qualitative and quantitative data will be collected during overlapping periods, analyzed independently, and integrated during interpretation [12]. The primary function of integration is complementarity: qualitative interview data will capture perceived experiences, mechanisms of benefit, and barriers to and facilitators of technical assistance engagement, whereas SNA and secondary productivity data will quantify collaboration patterns and research outputs.

The study sites encompass 10 academic institutions across multiple US regions, each serving as a coordinating center or hub providing IS support to NIH-funded EHE supplement projects.

### Evaluation Framework

**Figure 1:**
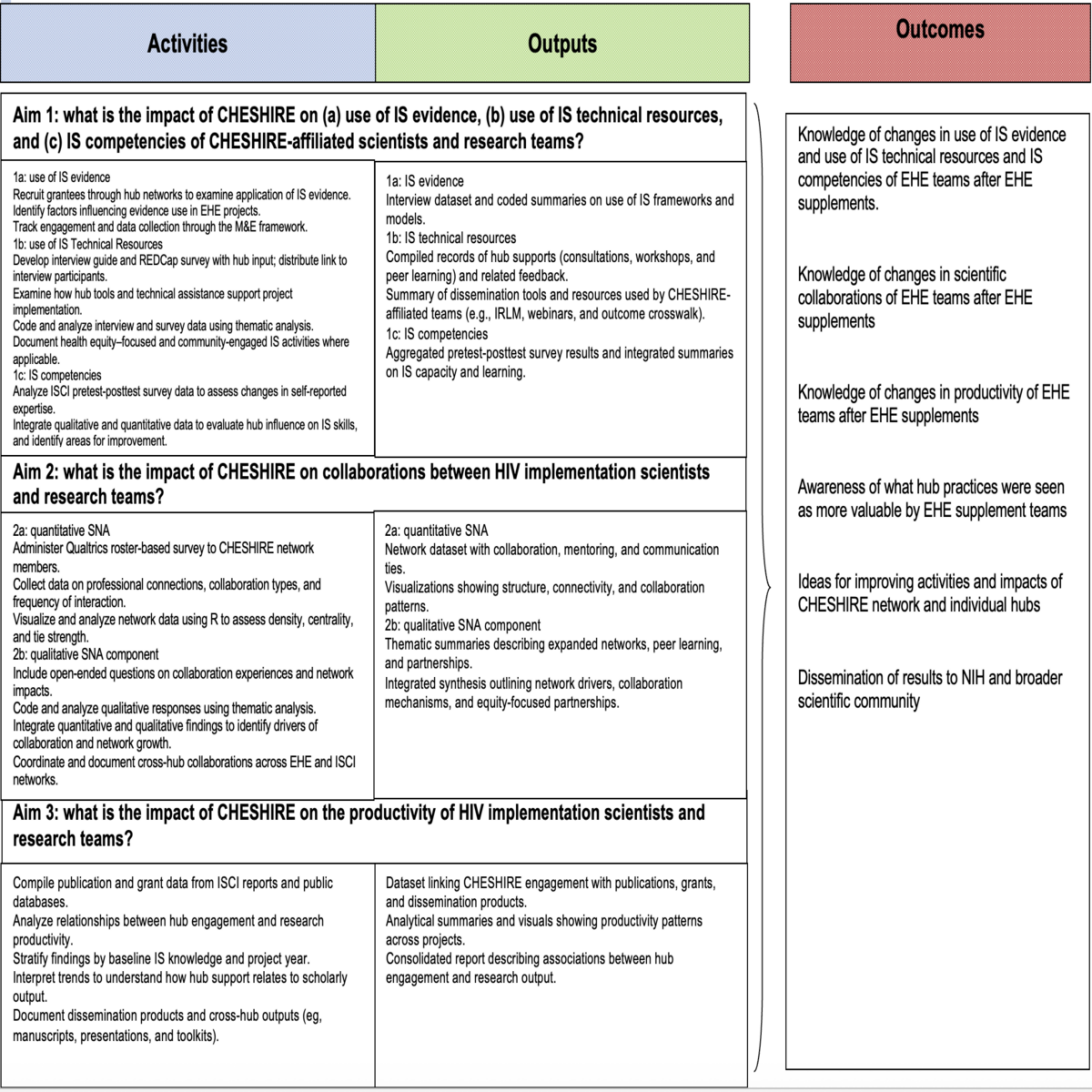
Evaluation activity flowchart for the network of Implementation Science Coordination Initiative (ISCI) and implementation science (IS) hubs. CHESHIRE: Capacity-Building Hubs to Enhance the Science of HIV Implementation Research; EHE: Ending the HIV Epidemic in the US; IRLM: implementation research logic model; M&E: monitoring and evaluation; NIH: National Institutes of Health; REDCap: Research Electronic Data Capture; SNA: social network analysis.

The evaluation framework identifies CHESHIRE activities, outputs, and outcomes and links each evaluation aim to corresponding framework components and outputs, shown in Figure 1. The framework guides the assessment of 3 domains: IS competencies and use of IS resources, scientific collaboration, and research productivity.

### Study Population

Participants include EHE-funded researchers receiving Center for AIDS Research (CFAR) and AIDS Research Center (ARC) supplements and CHESHIRE network hub affiliates, as previously described [6]. The evaluation population includes EHE project principal investigators and multiple principal investigators, IS hub investigators, mentors and coaches, coordinators, staff, and ISCI team members.

### Data Sources

**Figure 2:**
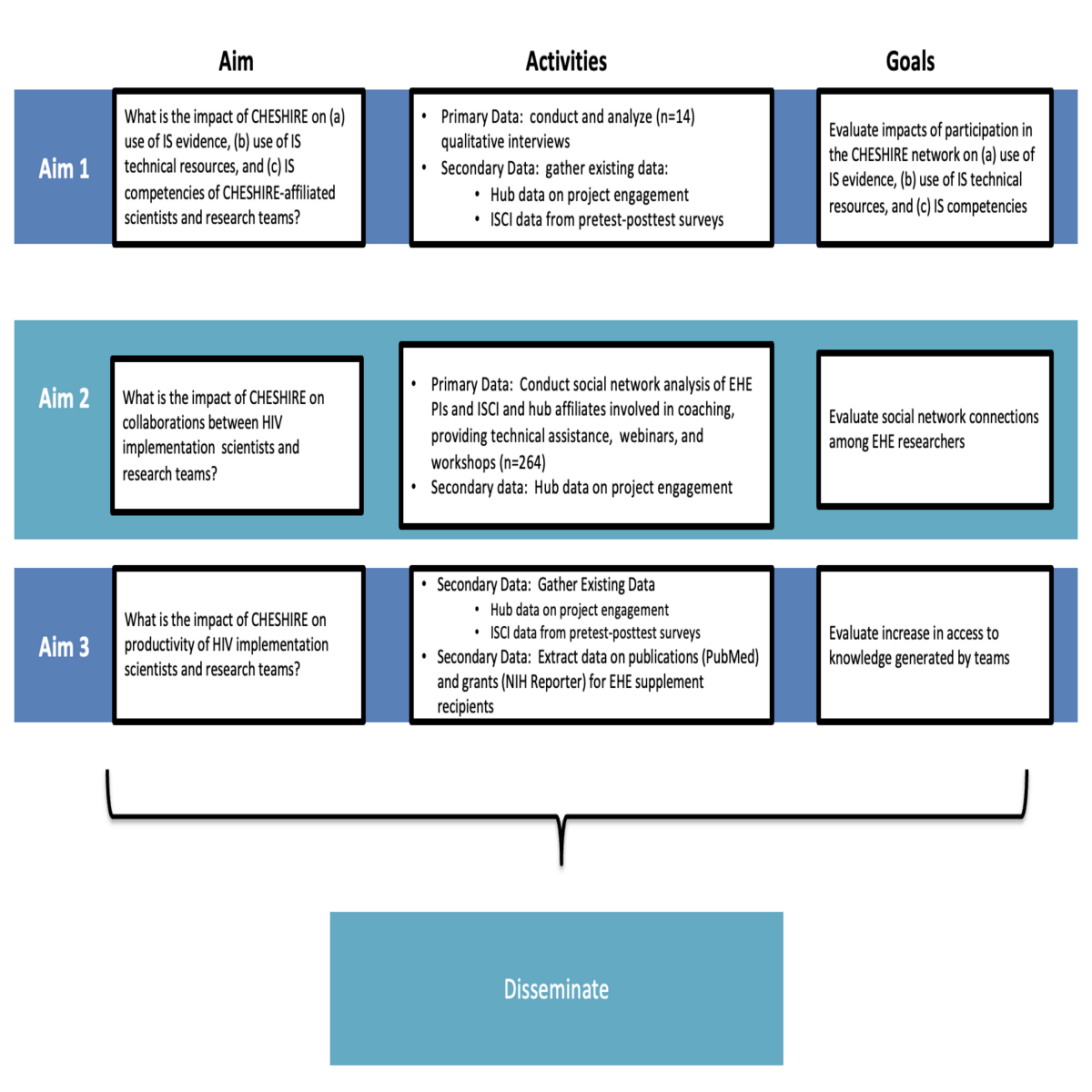
Data sources and goals. CHESHIRE: Capacity-Building Hubs to Enhance the Science of HIV Implementation Research; EHE: Ending the HIV Epidemic in the US; IS: implementation science; ISCI: Implementation Science Coordination Initiative; NIH: National Institutes of Health; PI: principal investigator.

We will draw on primary and secondary data sources to address the 3 evaluation aims (Figure 2). Primary data sources include qualitative interviews with EHE supplement leads and a Qualtrics (Qualtrics International Inc)-based SNA survey of CHESHIRE-affiliated individuals. Secondary data sources include ISCI EHE Project Final Progress Report Survey data, hub reports documenting hub-project interactions, NIH RePORTER data, PubMed and PubMed Central publication records, and ISCI progress report data. Table 1 summarizes key variables.

**Table 1:** Key variables considered for analysis.

Construct	Definition	Data source
IS^a^ competency	Self-perceived knowledge, skills, and confidence in applying IS principles, frameworks, and methods	Qualitative interviews (aim 1) ISCIb pretest-posttest survey (aim 3)
EHE^c^ researcher productivity	Scientific output in relation to EHE goals, including the number of publications, grants submitted or awarded, presentations, and other scholarly or practice-based contributions specific to the EHE initiative	ISCI EHE project final progress report (aim 3) PubMed (publications; aim 3) NIHd RePORTER (grants; aim 3)
Professional relationships	Network of collegial and collaborative connections that a researcher maintains within or outside their institution in relation to the EHE initiative	Qualitative interviews (aim 1) SNAe REDCapf survey (aim 2)
Frequency of interactions	How often a researcher engages with members of the IS hub	Qualitative interviews (aim 1) Hub data (aim 2)
Presence and type of EHE-related collaborations and mentorship	Joint projects between individual EHE projects or between EHE projects and hubs	Qualitative interviews (aim 1) SNA REDCap survey (aim 2)
Role	Hub affiliate or supplement lead or colead	SNA REDCap survey (aim 2)
Outside mentorship	Any mentoring relationships that the researcher maintains outside of the IS hub structure, particularly those that contribute to their career development, EHE research capacity, or IS skills	Qualitative interviews (aim 1) SNA REDCap survey (aim 2)
Wished-for collaboration	Unmet needs or desired support from the IS hub, particularly in forming collaborations, accessing expertise, or receiving resources that would enhance their capacity to conduct EHE-related IS research	Qualitative interviews (aim 1) SNA REDCap survey (aim 2)
Contributions to IS hub activities	Ways in which the researcher actively participates in and supports the activities of the IS hub	Qualitative interviews (aim 1) SNA REDCap survey (aim 2)

^a^IS: implementation science.

^b^ISCI: Implementation Science Coordination Initiative.

^c^EHE: Ending the HIV Epidemic in the US.

^d^NIH: National Institutes of Health.

^e^SNA: social network analysis.

^f^REDCap: Research Electronic Data Capture (Vanderbilt University).

Professional relationships assessed through the SNA survey are quantitative network variables, whereas professional relationships collected in qualitative interviews represent participants’ perceptions and experiences of collaboration. These sources will be integrated during interpretation to provide complementary perspectives rather than being treated as identical variables.

### Sampling and Recruitment

For qualitative interviews, we aim to recruit 4 supplement leads from each of the 9 hubs, including 2 high-engagement and 2 low-engagement projects per hub, for an anticipated total of 36 EHE projects. Engagement will be measured via the number of mentoring contacts between each project team and its hub mentoring team as collected in ISCI reports. We will contact supplement leads via email or telephone to invite them to participate in a semistructured interview.

For the SNA survey, we will invite 265 individuals to participate, including members of 9 IS hub teams (investigators, mentors and coaches, and staff) funded between 2019 and 2024 and principal investigators of EHE-related supplement projects awarded between 2019 and 2023. To populate the respondent list, IS hub and ISCI lead investigators and coordinators will provide the names, affiliations, and email addresses of investigators, mentors and coaches, and relevant staff on their teams. We will develop a contact list of supplement principal investigators using NIH publicly available data for each supplement grant. We will disseminate the Qualtrics survey via email and send reminders from the study team, IS hub leadership and coordinators, and CFAR and ARC leadership.

For secondary analyses of productivity, IS competencies, and hub engagement, we will include EHE projects with available ISCI EHE Project Final Progress Report Survey data, hub engagement data, NIH RePORTER data, PubMed and PubMed Central records, or ISCI progress report data as applicable to each aim.

### Data Collection Procedures

#### IS Knowledge and Competencies

The ISCI distributed pre- and postsurveys to EHE project teams before project initiation and after project completion. For this evaluation, we will analyze data from projects that completed the ISCI EHE Project Final Progress Report Survey between August 2021 and February 2025. Teams rated their current level of expertise as beginner, intermediate, or advanced for each of 10 implementation research competencies [13].

#### Hub Engagement

Hub-project interactions will be operationalized through frequency and satisfaction. Frequency of engagement will be measured using two sources: (1) the ISCI EHE Project Final Progress Report Survey item “What has the frequency of your engagement with your Hub been thus far,” with response options of “not enough,” “just right,” “too much,” and “not sure”; and (2) hub-reported counts of 1:1 technical assistance meetings between hubs and each project and other hub meetings attended by projects, such as communities of practice. Satisfaction will be measured using the ISCI EHE Project Final Progress Report Survey item “What is your overall satisfaction with working with your IS Hub so far?” with response options ranging from “very satisfied” to “very dissatisfied.”

#### Qualitative Interviews

Three trained interviewers will conduct interviews via Zoom (Zoom Video Communications) or Microsoft Teams. The interviewers will be unaffiliated with the hub of the interview participants. Interviews will last approximately 20 minutes. We will use the outcomes of capacity building identified in a recent systematic review [3], the CHESHIRE logic model, and the Behavior Change Wheel [14] to inform the interview guide. Questions will address capability, opportunity, motivation, and perceived impacts of the hub on the EHE supplement team. At the beginning of each qualitative interview, participants will complete a brief Likert survey via REDCap (Research Electronic Data Capture; Vanderbilt University) about their experience working with the hub to quantify their level of hub engagement. Interviews will be recorded on Microsoft Teams or Zoom and transcribed using automated technology. Interviewers will review transcripts for accuracy. Participants will not receive compensation for completing the interviews.

#### Social Network Survey

Respondents will be presented with a roster of invited individuals and asked to identify people whom they know professionally, defined as individuals with whom they have ever had direct professional interaction. For each professional contact, participants will indicate the types of scientific activities they engaged in with that contact before and after receipt of IS hub or EHE-related supplement funding and whether the postfunding activity was related to involvement in EHE supplements or IS hubs. Participants will also indicate frequency of contact with each professional contact. Answer options for scientific activity were adapted from previously used instruments and include (1) “I mentored this person in IS” (2) “This person mentored me in IS” (3) “We proposed research together” (4) “We collaborated on funded research” (5) “We presented research together” (6) “We co-authored research output” (7) “Other.” The IS acronym is defined in the survey; however, the survey does not provide a separate definition of “mentoring in IS.” Because respondents are IS hub team members or EHE supplement investigators assigned to receive hub services, we expect them to understand IS mentorship, but we acknowledge that respondents may conflate IS and non-IS mentorship. The survey will also include questions about the participants’ role within the CHESHIRE network, receipt of IS support from experts outside CHESHIRE, and level of IS expertise. The full survey instrument can be found in Multimedia Appendix 1.

#### Grant and Publication Data

Submitted grants will be identified from the ISCI EHE Project Final Progress Report Survey item “Have you applied for additional funding to continue work based on your EHE supplement project? Select all that apply.” Awarded grants will be identified from the survey item “From which of the applications selected above have you received additional funding to continue work based on your EHE supplement project? Select all that apply” and from NIH RePORTER searches. A member of the evaluation team will query NIH RePORTER for funded grants by EHE project principal investigators and multiple principal investigators and extract grant-related information into a Microsoft Excel file. The search for each principal investigator or multiple principal investigator will begin in the year after the EHE project was awarded. Two additional evaluation team members will review grant titles to ensure that the grant is related to HIV and/or IS.

Publications will be identified using PubMed Central and NIH RePORTER. Because EHE-associated publications should be linked to the parent CFAR or ARC, a member of the evaluation team will first look up each CFAR and ARC in NIH RePORTER. Second, the team will download all publications associated with each CFAR and ARC for each year since 2020. As the first EHE projects were awarded in 2019, we would not expect publications affiliated with a given project before 2020. Third, the team will scan the publication list for each CFAR or ARC to identify publications for which an EHE principal investigator or multiple principal investigator was an author, including first author, middle author, or last author. We will also confirm that the publication appeared after that specific EHE project principal investigator or multiple principal investigator received the EHE supplement. Two team members will review the final publication list to ensure that each publication is related to HIV and/or IS and meets the year criteria.

### Key Variables and Measures

Key variables include self-reported IS competency level for 10 implementation research competencies, frequency of and satisfaction with hub engagement, number of 1:1 technical assistance meetings, number of other hub meetings attended, qualitative perceptions of hub engagement and capacity building outcomes, professional network ties, type and frequency of scientific collaboration, receipt of IS support from outside CHESHIRE, role within the CHESHIRE network, submitted grant applications, awarded grants, publications, and baseline IS knowledge.

For EHE supplement principal investigators and multiple principal investigators, we will construct a variable indicating how often each supplement project engaged with IS hub services from its assigned hub using the hub engagement measures described above.

### Data Management

Survey, interview, network, and secondary data will be stored on secure, access-controlled systems. Interview transcripts will be reviewed for accuracy and deidentified before broader analytic review. Grant and publication abstraction files will be maintained in structured Microsoft Excel files and reviewed by multiple team members for eligibility and relevance. Only authorized members of the evaluation team will access identifiable data.

### Data Analysis

#### Aim 1: IS Competencies and Use of IS Resources

##### Data Source and Sample

Aim 1 will use ISCI EHE Project Final Progress Report Survey data, hub-reported engagement data, REDCap interview engagement surveys, and qualitative interviews with EHE supplement leads. The analytic sample for IS competency analyses will include projects with available pre- and postsurvey data for each competency. The qualitative sample will include up to 36 supplement leads selected to represent high- and low-engagement projects across hubs.

##### Variables and Collection Process

Variables include self-reported IS knowledge and competency level; frequency of and satisfaction with hub engagement; counts of 1:1 technical assistance contacts and other hub meetings; and qualitative descriptions of perceived benefits, barriers, facilitators, and mechanisms of hub support.

##### Analysis

For IS research knowledge and competencies, we will compare the proportions of projects by expertise level in the pre- and postsurveys and describe change in expertise level as “increase,” “no change,” or “decrease.” “Increase” will be defined as movement from beginner to intermediate or advanced or from intermediate to advanced; “decrease” will be defined as movement from advanced to intermediate or beginner or from intermediate to beginner. Analyses will be descriptive, and no formal power analysis was conducted because the evaluation is not designed for causal inference or hypothesis testing. We will repeat these analyses limiting the data to projects where increases or decreases were possible; for example, analyses of increases in IS knowledge will be limited to projects that reported beginner or intermediate knowledge at baseline because projects reporting advanced knowledge at baseline cannot increase their knowledge level.

Qualitative interview transcripts will be analyzed using ATLAS.ti (ATLAS.ti Scientific Software Development GmbH). We will use thematic analysis to identify patterns in participants’ experiences, focusing on engagement levels, perceived benefits, and barriers to and facilitators of effective use of IS resources. Three research team members, 2 of whom will conduct the interviews, will create a coding framework a priori and then separately apply the coding framework to each transcript. They will compare applied codes and resolve differences before continuing.

##### Limitations

Aim 1 analyses rely on self-reported competency ratings and satisfaction measures and will be interpreted descriptively. Interview findings may be influenced by social desirability bias, although the recruitment strategy intentionally includes both high- and low-engagement projects to capture diverse experiences.

#### Aim 2: Scientific Collaboration Networks

##### Data Source and Sample

Aim 2 will use the SNA survey of 265 invited CHESHIRE-affiliated individuals, including IS hub team members, ISCI members, and EHE supplement principal investigators.

##### Variables and Collection Process

Network variables include professional ties, type of scientific activity, timing of activity relative to IS hub or EHE-related supplement funding, whether the activity was related to EHE supplement or IS hub involvement, and frequency of contact. Additional variables include CHESHIRE role, receipt of IS support from experts outside CHESHIRE, and level of IS expertise.

##### Analysis

We will describe current collaboration networks and assess change after CHESHIRE involvement using network metrics and exponential random graph modeling. The SNA will assess changes in density and centrality of professional connections among CHESHIRE participants. We will visualize collaboration networks, calculate network descriptive statistics, and use exponential random graph modeling to build and test predictive models of network ties among EHE projects and IS hubs [15-17]. We will use the *statnet*, *igraph*, and *ergm* packages in the R statistical software (version 4.3.0; R Foundation for Statistical Computing) to conduct network analyses.

##### Limitations

Because this evaluation does not include an unexposed comparison group, network findings will be interpreted as changes observed following CHESHIRE implementation rather than causal impacts attributable solely to the CHESHIRE network. In addition, respondents may conflate IS and non-IS mentorship when reporting mentorship ties.

#### Aim 3: Research Productivity

##### Data Source and Sample

Aim 3 will use ISCI final reports, NIH RePORTER data, PubMed and PubMed Central records, and ISCI EHE Project Final Progress Report Survey data. The analytic sample will include EHE projects with available data among the 248 total EHE projects.

##### Variables and Collection Process

Productivity variables include submitted grant applications, awarded grants, and peer-reviewed publications linked to EHE project principal investigators or multiple principal investigators. Grant and publication data will be abstracted and reviewed by the evaluation team as described above.

##### Analysis

Longitudinal tracking of grant submissions and publications will describe research productivity following CHESHIRE implementation. Aim 3 productivity analyses will be stratified by baseline IS knowledge captured in the baseline survey and separately reported for projects with beginner, intermediate, and advanced IS knowledge.

##### Limitations

Because the study has no control or comparison group, productivity analyses will not be interpreted as causal estimates of CHESHIRE impact. Publication and grant capture may be incomplete if outputs are not linked to the parent CFAR or ARC, are not indexed in the queried sources, or are not identifiable from available project records.

### Mixed Methods Integration

Qualitative and quantitative findings will be integrated during interpretation [12,18]. Interview data will help explain how and why hub engagement may influence capability, opportunity, motivation, use of IS resources, perceived capacity, and collaboration. Quantitative SNA and productivity analyses will provide descriptive evidence of collaboration patterns and research outputs following CHESHIRE implementation. Integration will focus on convergence, complementarity, and divergence across data sources to generate a more complete understanding of CHESHIRE outcomes than any single method could provide.

### Missing Data and Bias Considerations

Missing survey data will be described for each data source and variable. For descriptive pretest-posttest comparisons of IS knowledge and competencies, analyses will be limited to projects with available baseline and follow-up responses for each competency. Potential sources of bias include self-reported competency ratings, social desirability bias during interviews, incomplete capture of publications or grants, differential SNA survey response, and the absence of a control or comparison group. Additional confounders may include prior IS experience, institutional resources, funding level, and baseline productivity. These limitations will be considered when interpreting the findings.

### Ethical Considerations

The evaluation involves survey, interview, network, and secondary data sources and will be conducted in accordance with applicable institutional review board requirements. Research team members from each institutional team prepared their own institutional review board applications drawing on a template from the protocol chair (Yale University) to align with their institutional guidelines and requirements. Three institutions determined that the evaluation was not human subject research: the University of Washington (STUDY00020987), Johns Hopkins University, and Northwestern University. The other 7 institutions determined that the evaluation was exempt from ethical review: University of California, Los Angeles (24-000984); University of California, San Diego (810934); Yale University (2000038229); University of Alabama at Birmingham (IRB-300013406-002); Vanderbilt University (241155); University of Texas Health Science Center at Houston (HSC-SPH-24-0856); and Emory University (STUDY00008340).

Interview participants will be provided with information about the study before they take part. To strengthen qualitative rigor and address evaluator positionality, interviewers will not be affiliated with the institutions that provided mentorship to the participants. Interviews will be conducted by staff who did not deliver mentorship or technical assistance to EHE grantees. Individuals with a prior relationship with a participant will not lead that participant’s interview or access interview materials before deidentification.

## Results

The CHESHIRE coordinating center and hubs were funded from 2020 to 2025 through supplements to NIH-funded CFARs and National Institute of Mental Health–funded ARCs, as stated in the Funding section.

The study sampling frame included principal investigators and multiple principal investigators of EHE supplement projects funded between 2019 and 2023 (all analyses), as well as IS hub and ISCI team members (SNA only). The master list started with 280 EHE supplement multiple principal investigators and hub team members, of whom 5 (1.8%) were ineligible, 2 (0.7%) were deceased, and 8 (2.9%) were unreachable. A total of 265 network members were in the final recruiting list, including 205 (77.4%) supplement grantees only, 18 (6.8%) dual-role members (both grantees and hub and ISCI team members), and 42 (15.8%) hub and ISCI team members only. As of January 2026, we have completed enrollment. Data abstraction and analysis are currently ongoing, and evaluation findings will be disseminated after completion. The anticipated submission of the protocol results is in December 2026.

We anticipate that CHESHIRE participation will be associated with enhanced researcher IS competencies, stronger collaborations, and increased research productivity. Key outcomes include (1) increases in self-reported IS competencies among EHE researchers, as indicated by changes in pre- and postsurvey data; (2) strengthened professional connections among HIV implementation scientists across hubs, demonstrated through network expansion and higher collaboration frequency; (3) an increase in grant applications and funded projects among EHE-affiliated teams, reflecting an increased ability to secure funding for IS-related research; (4) increased productivity and scientific contributions, measured via peer-reviewed publications; and (5) identification of key facilitators of and barriers to effective IS capacity building, providing actionable recommendations for optimizing CHESHIRE’s support strategies.

The findings will contribute to refining technical assistance models for IS capacity building and guiding the NIH’s future investment in collaborative research networks. We anticipate that the NIH will use identified best practices to improve cross-hub coordination and optimize technical support models. The study will provide empirical evidence on the effectiveness of CHESHIRE’s structured approach to capacity building and inform policy development for future large-scale IS initiatives.

Expected outcomes of the network activities include stronger IS in the field of HIV research and practice and stronger dissemination of IS results within the research and practice communities.

## Discussion

### Expected Findings

Several recent studies of capacity-building initiatives have identified a need for evaluation capacity to assess impacts [19]. Within the field of global agriculture, a recent study compared approaches to evaluation capacity building across 4 agricultural research organizations and highlighted the need for evaluation processes that organically assess interacting dimensions of the relevant impact pathways [20]. Global health capacity-building initiatives were assessed in another recent study, and authors recommended the development and use of more standardized evaluation methods [21]. While we do not have a baseline, we will be able to compare our SNA results with those of other collaborations, such as the Implementation Science Centers in Cancer Control. A systematic review of dissemination and IS capacity-building programs worldwide highlighted the need for harmonized reporting and evaluation measures to strengthen cross-program comparisons [22], as did a recent mixed methods study of 3 dissemination and IS capacity-building programs at one university exploring the functions and forms of consultation activities [23].

There is emerging literature on evaluating capacity-building initiatives using a range of methods. A recent study evaluated a knowledge translation capacity-building initiative using pretest-posttest surveys of participating occupational therapy clinicians who reported significant improvements in knowledge, skills, and beliefs about their own capabilities as well as their experience that knowledge translation had been integrated into organizational culture [24]. A 2020 SNA of new collaboration networks for the Implementation Science Centers in Cancer Control was conducted to establish a baseline for future comparisons to assess the impact of the collaborative network on collaboration activities [25].

This study addresses a critical gap in evaluating large-scale IS training initiatives [26-28]. By assessing outcomes following CHESHIRE implementation, we can examine whether its hub-based model is an effective strategy for scaling IS capacity and fostering interdisciplinary collaborations [29,30]. Qualitative findings will provide insights into the mechanisms and contextual factors underlying observed evaluation outcomes, including how hub engagement may influence capability, opportunity, motivation, perceived capacity, and use of IS resources. If the findings are favorable, this model may inform adaptations in other public health domains requiring rapid deployment of implementation strategies.

Challenges in evaluating network-wide initiatives include varying levels of engagement, differences in regional priorities, and limitations in capturing long-term impacts. Additional limitations include the absence of a control or comparison group; limited ability to account for confounders such as prior experience, institutional resources, funding level, and baseline productivity; and the possibility that respondents may have interpreted IS mentorship differently in the SNA survey. To mitigate these challenges, the study integrates multiple data sources, stratifies productivity analyses by baseline IS knowledge, and includes qualitative procedures designed to reduce social desirability and insider status bias. The findings will guide improvements in IS research networks and inform scalable strategies for IS technical assistance beyond the EHE initiative.

### Conclusions

By describing outcomes following CHESHIRE implementation, this study aims to enhance IS capacity, facilitate collaboration, and improve HIV research implementation strategies. The findings will inform policy recommendations for strengthening IS support structures in public health initiatives. The evaluation will contribute to broader efforts to optimize technical assistance models in federally funded implementation research networks. Future directions will include longitudinal studies to examine the sustainability of CHESHIRE’s impact and potential adaptations for networks servicing efforts to address broader public health challenges.

## Appendix

Multimedia Appendix 1 Social network analysis survey.

## Abbreviations

ARC: AIDS Research Center
CFAR: Center for AIDS Research
CHESHIRE: Capacity-Building Hubs to Enhance the Science of HIV Implementation Research
EHE: Ending the HIV Epidemic in the US
IS: implementation science
ISCI: Implementation Science Coordination Initiative
NIH: National Institutes of Health
REDCap: Research Electronic Data Capture
SNA: social network analysis
