# Supplementary material for: Evaluating the Outcomes of the Capacity-Building Support From a Collaborative Network of Implementation Science Technical Assistance Hubs: Protocol for a Mixed Methods Evaluation
Source: JMIR Res Protoc. 2026 Jul 17;15:e91374. doi: 10.2196/91374 (PMC13428205; doi:10.2196/91374)
Supplement: Multimedia Appendix 1 [file resprot_v15i1e91374_app1.docx]

**Welcome to the EHE Implementation Science Hub Collaborations Survey!**

[Insert IRB-approved consent form.]

**1. What have been your roles to date in relation to the EHE-funded supplements and associated Implementation Science (IS) Hubs?** Please select all that apply.

- I lead/led or co-lead/co-led an EHE-funded supplement project
- I am a member of an IS Hub team, providing technical assistance, coaching, and other services to EHE-funded projects
- I am/was a member of the ISCI team
- Other __________________________________________________

**2. From the list below, whom do you know professionally? Please indicate people with whom you have ever had direct professional interaction**, i.e., in-person or online conversations and meetings, phone calls, emails. We are asking about all professional interactions, not just those related to EHE supplement grants or IS Hubs.

Please do not count listservs, mass emails, or attendance of webinars as direct interaction.

This list consists of EHE supplement MPIs, IS Hub teams, and the ISCI team, and is organized alphabetically. Please feel free to add anyone else in this group who is not listed and whom you know professionally.

*Using CTRL + F or COMMAND + F will allow you to search the list for names. Please do not select yourself.*

** IS Hub team member*

- Name
- Name
- … etc
- Did we miss anyone? Enter here: ____________________

**Now we will ask about the type of scientific relationship you have with the individuals you selected earlier.**

**3. Prior to your involvement in an EHE supplement, IS Hub, or ISCI, what scientific activities had you engaged in with each person?** Please select all statements that are true for your relationship with each person listed.

*For each person, select all activities that apply. If none apply, please leave blank.*

*Please be sure to scroll to the right to see all columns.*

|  | I mentored or coached this person in **IS** | This person mentored or coached me in **IS** | I received formal training from this person **in IS** | We planned a research activity together | We submitted a proposal for research funding | We collaborated on a research project | We presented together at a conference or meeting | We wrote a scientific paper together | Other |
| --- | --- | --- | --- | --- | --- | --- | --- | --- | --- |
| Person 1 |  |  |  |  |  |  |  |  |  |
| Person 2 |  |  |  |  |  |  |  |  |  |
| Person 2 |  |  |  |  |  |  |  |  |  |
| Person 4 |  |  |  |  |  |  |  |  |  |

**If selecting ‘Other’ above, please explain:**

_________________________________________________________

**4. Since the start of your involvement in an EHE supplement, IS Hub, or ISCI, what scientific activities had you engaged in with each person?** Please select all statements that are true for your relationship with each person listed.

For each true statement (“Yes”), please indicate if any part of the activity was related to your involvement in the EHE supplements or IS Hubs ("EHE-related").

*For each person, select all activities that apply. If none apply, please leave blank.*

*Please be sure to scroll to the right to see all columns.*

|  | I mentored or coached this person in **IS** | This person mentored or coached me in **IS** | I received formal training from this person **in IS** | We planned a research activity together | We submitted a proposal for research funding | We collaborated on a research project | We presented together at a conference or meeting | We wrote a scientific paper together | Other |
| --- | --- | --- | --- | --- | --- | --- | --- | --- | --- |
| Person 1 | - Yes - EHE-related | - Yes - EHE-related | - Yes - EHE-related | - Yes - EHE-related | - Yes - EHE-related | - Yes - EHE-related | - Yes - EHE-related | - Yes - EHE-related | - Yes - EHE-related |
| Person 2 | - Yes - EHE-related | - Yes - EHE-related | - Yes - EHE-related | - Yes - EHE-related | - Yes - EHE-related | - Yes - EHE-related | - Yes - EHE-related | - Yes - EHE-related | - Yes - EHE-related |
| Person 2 | - Yes - EHE-related | - Yes - EHE-related | - Yes - EHE-related | - Yes - EHE-related | - Yes - EHE-related | - Yes - EHE-related | - Yes - EHE-related | - Yes - EHE-related | - Yes - EHE-related |
| Person 4 | - Yes - EHE-related | - Yes - EHE-related | - Yes - EHE-related | - Yes - EHE-related | - Yes - EHE-related | - Yes - EHE-related | - Yes - EHE-related | - Yes - EHE-related | - Yes - EHE-related |

**If selecting ‘Other’ above, please explain:**

_________________________________________________________

**5. In the last year, how often did you interact with each person directly?** Direct interactions include in-person or online conversations and meetings, phone calls, emails.

|  | Never | Once | A few times | Monthly | Weekly | Daily |
| --- | --- | --- | --- | --- | --- | --- |
| Person 1 |  |  |  |  |  |  |
| Person 2 |  |  |  |  |  |  |
| Person 3 |  |  |  |  |  |  |
| Person 4 |  |  |  |  |  |  |
| Etc. |  |  |  |  |  |  |

**6. Are there individuals in the EHE-grantee/IS Hub network that you would have liked to connect or collaborate with, but you did not have the opportunity to do so?**

- No
- Yes, please write their name(s) here: ________________________________________________

**7. Have you received IS-related mentorship or advice from anyone outside of the EHE supplement / IS Hub network during your involvement with the network?**

- No
- Yes, please write their name(s) here: ________________________________________________

**8. How would you rate your level of expertise in conducting dissemination and implementation research?**

- Beginner
- Intermediate
- Advanced

**9. Which of these IS Hub research efforts did you contribute to?**

- [List of activities]
- Other (please fill in): _______________________

**10. Please share any additional connections or collaborations that you have developed as a result of your participation in the** **EHE supplement grant, IS Hubs, and ISCI network.**  .

_____________________________________________________

_____________________________________________________

_____________________________________________________

**11. We would love to hear about any specific experiences, opportunities, or insights related to this network that have enriched your professional or personal life.** Please describe any specific stories/experiences.

________________________________________________________________

________________________________________________________________

________________________________________________________________

________________________________________________________________

________________________________________________________________

**We thank you for your time spent taking this survey. Your response has been recorded.**
